# Leveraging the role of community pharmacists in the prevention, surveillance, and treatment of opioid use disorders

**DOI:** 10.1186/s13722-019-0158-0

**Published:** 2019-09-02

**Authors:** Paxton Bach, Daniel Hartung

**Affiliations:** 1 British Columbia Centre on Substance Use, University of British Columbia, 400-1045 Howe Street, Vancouver, BC V6Z 2A9 Canada; 2 College of Pharmacy, Oregon State University/Oregon Health and Science University, Robertson Collaborative Life Science Building, 2730 SW Moody Ave, CL5CP, Portland, OR 97201-5042 USA

**Keywords:** Community pharmacy services, Opioid-related disorders, Opiate substitution treatment, Harm reduction

## Abstract

The global rise in opioid-related harms has impacted the United States severely. Current efforts to manage the opioid crisis have prompted a re-evaluation of many of the existing roles in the healthcare system, in order to maximize their individual effects on reducing opioid-associated morbidity and preventing overdose deaths. As one of the most accessible healthcare professionals in the US, pharmacists are well-positioned to participate in such activities. Historically, US pharmacists have had a limited role in the surveillance and treatment of substance use disorders. This narrative review explores the literature describing novel programs designed to capitalize on the role of the community pharmacist in helping to reduce opioid-related harms, as well as evaluations of existing practices already in place in the US and elsewhere around the world. Specific approaches examined include strategies to facilitate pharmacist monitoring for problematic opioid use, to increase pharmacy-based harm reduction efforts (including naloxone distribution and needle exchange programs), and to involve community pharmacists in the dispensation of opioid agonist therapy (OAT). Each of these activities present a potential means to further engage pharmacists in the identification and treatment of opioid use disorders (OUDs). Through a careful examination of these approaches, we hope that new strategies can be adopted to leverage the unique role of the community pharmacist to help reduce opioid-related harms in the US.

## Introduction

North America is currently in the midst of an opioid crisis that claimed nearly 70,000 lives in the US in 2018 alone [1]. Despite the existence of effective, evidence-based treatment strategies to treat patients with opioid use disorder (OUD), a majority of those with active OUDs are still not engaged in any form of treatment [2]. Increasing efforts are now being made to improve access, but a variety of persistent social, legal, and geographic reasons continue to limit entry into treatment for a significant portion of the US population.

In recent times, we have seen a number of additions to the pharmacologic armamentarium of medications to treat OUD, including extended-release formulations of naltrexone and buprenorphine. National efforts have also been made to expand access to prevention and treatment programs, such as the declaration of a national public health crisis in 2017, and the passage of several important pieces of federal legislation including the Substance Use-Disorder Prevention that Promotes Opioid Recovery and Treatment (SUPPORT) for Patients and Communities Act [3–5]. In spite of these efforts, the death toll is continuing to rise [1]. Though the causes of the opioid crisis are complex, it is clear that the present situation demands a broader re-evaluation of both how and where we provide care for patients with OUDs.

Historically, the treatment of substance use disorders has emphasized the role of specialist physicians and psychiatrists, often operating out of designated addiction treatment facilities. While effective for individual patients, this model does little to break down geographic barriers that make accessing addiction treatment particularly difficult for rural and/or marginalized populations. Designated clinics may also inadvertently continue to propagate the stigma around substance use disorders, a significant and ongoing social barrier in the provision of treatment. In an effort to expand access to OUD treatment, primary care clinicians, including nurse practitioners and physician assistants, are increasingly engaged in providing office-based opioid agonist treatment. Pharmacies represent a unique and relatively untapped resource in the fight against the opioid crisis—one which has been leveraged with some success in countries other than the United States. With highly trained staff, widespread distribution, and a high degree of public trust expressed towards pharmacists in the US, they may present an opportunity for clients with OUD to make contact with a healthcare professional in a setting that is much more abundant than designated clinics [6].

In this narrative review, we explore the evidence behind a number of strategies whereby community pharmacies and pharmacists can be engaged in the prevention, identification, and management of patients with OUD. Beginning with screening approaches to identify at-risk patients, we move on to discuss opportunities for patient education, strategies for the implementation of harm reduction programs, and pharmacy-based treatment programs. Some of these systems are experimental in nature, while others have a proven track record of effectiveness in other parts of the world. In learning from experiences both within the US and abroad, valuable new approaches may be adopted to help manage the devastating harms that continue to be wrought by the ongoing overdose epidemic.

## Pharmacists’ role in prescription opioid screening, management, and education

Pharmacists have extensive training related to the therapeutic use of pharmaceuticals. In the normal course of practice, community pharmacists continuously evaluate the appropriateness of medication use, monitor for potentially risky uncoordinated care, and check for drug interactions. With over 89% of the US population within 5 miles of a pharmacy, pharmacists are also among the most readily accessible healthcare professionals in the community and can play a valuable role in many primary healthcare functions [7]. In 2018, retail pharmacies in the US dispensed 169 million opioid prescriptions [8]. Unsurprisingly, pharmacies and the healthcare system continue to be a major source of diverted opioids [9]. Because pharmacists are typically the final healthcare professional patients encounter before using prescription medications, they are well positioned to screen for diversion, monitor for potentially problematic use of prescription opioids, and educate patients about opioid-related risks. Given that opioid prescriptions dispensed from pharmacies continue to be a central feature of the opioid crisis, there are compelling reasons why pharmacists should be involve in reducing prescription opioid-related health risks.

Reviewing prescriptions for safety and appropriateness is a core legal and clinical responsibility for the community pharmacist. At a minimum, pharmacists are legally required to ensure that controlled substance medication prescriptions have a legitimate medical purpose and are not diverted [10]. This commonly involves screening for potentially forged or altered prescriptions. Additionally, pharmacists are required to be vigilant for behavior that suggests diversion such as fills at multiple pharmacies, early fills, cash payments, or prescriptions from several prescribers. These behaviors, however, may also indicate the presence of an underlying OUD. A great challenge that pharmacists face is the need to balance their legally required role to monitor for diversion and other aberrant behavior with their clinical caregiving responsibilities to provide safe, effective, and timely access to treatments. These challenges are only intensifying as states, payers, and other healthcare entities increasingly impose restrictive opioid prescribing policies that often fall to the pharmacy profession to administer. Education and open communication among both pharmacy and prescriber communities are essential to striking a balance to avoid disruption of appropriate patient care.

Because pharmacists often interact with patients who may have undiagnosed OUD, there is growing interest in developing the capacity of the profession to provide interventions or direct treatment referrals for individuals who may have an OUD. In particular, the screening, brief intervention (SBI), with or without Referral to Treatment (SBIRT) models are evidence-based approaches to identify and reduce risks associated with problematic use of drugs and alcohol. The model has been deployed successfully in a variety of clinical and community settings. Surveys suggest that community pharmacists are strongly interested in helping patients with OUD, and have positive attitudes about pharmacy-based screening and intervention activities [11, 12]. Several studies have established the feasibility and acceptability of delivering brief alcohol interventions in community pharmacies in the UK, although effectiveness on risky alcohol drinking is less clear [13–15]. There is also evidence that patients are receptive to brief educational interventions on opioid safety and overdose prevention when delivered by pharmacists in the emergency room [16]. Implementation of SBI type interventions by community pharmacists are only beginning to be described and there are a number of efforts underway examining different models screening and intervention delivered through community pharmacies [17–20]. Resources to assist with referral to treatment are widely available, including the treatment services locator provided by SAMHSA (https://www.samhsa.gov/find-treatment).

In many states and healthcare systems (e.g. US Department of Veterans Affairs, state authorized collaborative practice agreement models), pharmacists have expanded authority to coordinate drug therapy management for chronic conditions [7]. Within these systems, pharmacists often collaborate with other in the healthcare system to partner with others in the healthcare system to monitor and manage a variety of chronic conditions; screening for signs of OUD and potentially referring patients for treatment is a natural extension of many of these existing clinical duties. Practice models that harness the unique skills and position of community pharmacists to identify and intervene with patients at high-risk for, or unrecognized, OUD have started to emerge [17, 18, 21, 22]. A pilot study by Strand et al. [21] describes a pharmacy-based screening tool and algorithm to identify and provide care for individuals who may be at high-risk for misuse and opioid-related harms. In this study, community pharmacists used an opioid risk prevention tool kit to identify patients at high risk for misuse and provide a structured approach for communicating with prescribers and offering different risk mitigation services such as naloxone. While the study sample was modest (n = 107) and derived from one state (North Dakota), 30% of screened individuals were identified by pharmacist as having an elevated risk for overdose and were provided services to reduce this risk. Although preliminary, these data suggest community pharmacist are capable of integrating screening tools into their practice to identify individuals at high risk for opioid-related harms.

Pharmacists often report a lack of information and clinical connection to other healthcare professionals as a barrier to providing more comprehensive and careful oversight of prescription opioid use [23, 24]. Most community pharmacists do not have access to medical records which further hinders their ability to gain a comprehensive understanding of patient circumstances. The emergence and accessibility of prescription drug monitoring program (PDMP) provide pharmacists with additional information with which to monitor for diversion and assess opioid-related risk [25]. However, similar to other healthcare professionals, pharmacists face considerable barriers effectively integrating PDMP reports into their practice [26, 27]. PDMP functionality has been a critical barrier to efficient use. There are efforts underway to enhance the user interface and integrate within dispensing systems and other types of decision support software. In our own work (pharmacistrespond.org), we have developed education and tools to provide community pharmacist with resources to raise awareness, improve how pharmacists use PDMP resources, and enhance communication between patients and providers.

Outside integrated health systems, community pharmacists can leverage other models to expand their clinical role to identify and care for individuals with OUD. In particular, medication therapy management (MTM) is an existing clinical practice framework that has the potential to be adapted and directed toward individuals with or at risk for OUD. MTM payment and practice model authorized under the 2003 Medicare Modernization Act that requires Medicare Part D plans to develop programs to optimize medication use and reduce risks of adverse events among beneficiaries with chronic conditions and who take multiple medications. Specifically, Part D MTM programs are designed to target specific patterns of medication-related problems (e.g. adherence) among individuals with core chronic conditions including hypertension, diabetes, heart failure, and dyslipidemia. Importantly, the MTM model includes a framework that allows for pharmacists to bill for these medication-related services. Notably, provisions (section 6064) of the recently passed Substance Use-Disorder Prevention that Promotes Opioid Recovery and Treatment (SUPPORT) for Patients and Communities Act advance the capacity of pharmacists to provide MTM services to individuals at risk for addiction.

Finally, patient education is also a core function for pharmacists. Educating patients about opioid safety risks should be a natural extension of this practice. There are numerous ways that pharmacists can educate patients about opioid-related safety such as discussing side effects, legal requirements for refills, and proper use, storage, and disposal. A large proportion of prescription opioids that are misused are obtained from friends or relatives [28]. A recent survey found that one in five adults reported sharing opioid medications with other individuals [29]. Another study found that opioid prescriptions to family members increased the risk of overdose among individuals who were not directly prescribed an opioid [30]. Unfortunately, nearly half of all patients who receive prescription opioids do not report receiving information about disposal. Of those that do, only one-third report receiving this information from the pharmacist [29]. Pharmacies play an important role educating patients about these risks and providing information about secure storage and disposal options. Pharmacies in the US can now register with the Drug Enforcement Agency to become a registered collection site for unused medications. In 2018, Washington passed legislation to develop the first statewide drug takeback program to facilitate collection of and disposal of unused medications in the community, and a number of other states have since followed suit [31].

## Naloxone distribution from community pharmacies

One of the most promising and tangible pharmacist activities to reduce opioid-related risks has been through increasing the distribution of naloxone. Enhanced distribution of naloxone has been a core strategy directed at the opioid epidemic [32]. Naloxone has historically been distributed through community-based naloxone distribution programs and used by first responders [7]. There are several studies showing that naloxone distribution programs reduce overdose risks [33]. Although naloxone has been available directly to patients with a prescription, recent legislative and regulatory changes have made it easier for individuals obtain naloxone through pharmacies. These changes include laws providing legal immunity for prescribers and dispensers of naloxone, third-party prescriptions that allow naloxone to be prescribed to someone who is not themselves at risk for overdose, standing order or collaborative practice agreements that allow pharmacies to dispense without a prescription from a provider, and pharmacist prescriptive authority [34]. Currently, naloxone is available without a patient-specific prescription from another medical professional in all 50 states and the District of Columbia. Pharmacists are often in an ideal position to identify individuals who are at an elevated risk for opioid overdose and provide naloxone directly (e.g. individuals using high dose opioids, co-prescribed benzodiazepines, receiving treatment for OUD, etc.) [35].

Passage of naloxone access laws have significantly increased naloxone dispensing in the retail pharmacy setting. Between 2007 and 2016, the number of naloxone prescriptions in retail pharmacies increased nearly 100-fold, from 1488 to 147,457 prescriptions [36]. Emerging evidence suggests that expansion of naloxone access laws is also associated with reductions in opioid-related overdose fatalities [37, 38]. A study by McClellan et al. [38] found that between 2000 and 2014, states implementing naloxone access laws experienced 14% fewer opioid overdose deaths. Notably, another recent study found states with more expansive naloxone access laws that granted direct authority to pharmacists to dispense naloxone had the largest effect on naloxone dispensing and opioid-related deaths [37]. Despite these encouraging data, the frequency of naloxone dispensing remains relatively low given the magnitude of the overdose epidemic. A study in California, which allows naloxone to be dispensed under a Board of Pharmacy protocol, found that only 24% of surveyed pharmacies indicated they were able to provide naloxone without a prescription. Further, a non-trivial number of pharmacies provided erroneous information, such as recommending a buprenorphine–naloxone product not indicated to reverse overdose [39]. In another study, only a third of pharmacies in Philadelphia, where a state-wide naloxone standing order had been in place for more than 3 years, were able to provide nasal naloxone without a prescription [40]. A survey of community pharmacists in Indiana, where access laws are similar, found less than half of pharmacists were comfortable dispensing naloxone [41]. While a number of states and municipalities have established successful pharmacy-based naloxone distribution programs, the totality of evidence suggests a great need for pharmacist training and education related to naloxone, both within schools of pharmacy but also for practicing pharmacists [42].

## Pharmacy-based harm reduction strategies

The term “harm reduction” refers to any approach designed to minimize the negative consequences associated with a specific behaviour, without necessarily targeting the behaviour itself [43]. When applied to substance use disorders, it encompasses a number of evidence-based approaches that have been shown to reduce negative health and social outcomes, including decreased transmission of bloodborne infections and prevention of overdose deaths [44]. The strategic employment of harm reduction approaches also has the potential to offer opportunities for some of our most vulnerable patients to engage further with the healthcare system [45]. A number of studies have examined the implementation of harm reduction strategies into the community pharmacy environment, both within the US and abroad.

One of the most studied forms of harm reduction in the US has been initiatives to increase access to clean needles for people who inject drugs intravenously. This idea was borne out of the HIV epidemic in the mid-1990s, where available evidence suggested it could be an effective approach in limiting the spread of HIV among people who inject drugs [46]. The least resource-intense means of accomplishing this has been to change in state laws which prohibited the sale of non-prescription syringes in private pharmacies. Where enacted, such a policy change has been associated with a decrease in syringe sharing behaviour, but results have been highly variable due largely to a lack of clarity in the individual state laws and varied uptake by pharmacies [47].

Although pharmacist generally agree that there are public health benefits of making syringes non-prescription, attitudes about the role of pharmacies and pharmacists vary [47]. The most common concern voiced by pharmacists is one of concern for their staff and facilities, though data have suggested no measurable increase in crime or substance use in the area surrounding pharmacies that have adopted this policy [48, 49]. Among people who inject drugs, their experiences using pharmacies to purchase clean syringes has also varied widely, though in general attitudes have been positive, particularly among females [47]. Despite these results, there remains considerable heterogeneity among state laws around the non-prescription sale of injecting equipment. Some states specifically authorize the sale, while others do not have a law that directly prohibits sale to people who inject drugs, while others still have a ban in place for syringe sale to people who inject drugs [50]. The lack of a clear, consistent understanding of the rules governing this type of intervention has been identified as a key limitation in its widespread uptake [47].

While expanding the sale of non-prescription syringes is a widely adaptable intervention, the cost of individual syringes and the quantities they must be purchased in can still present a barrier to the most vulnerable clients [47, 51]. An alternative approach to increasing access to clean syringes has been the establishment of designated needle/syringe exchange programs. The data on the effectiveness of these strategies suggest that they are effective at reducing transmission of HIV and hepatitis C virus (HCV), especially when paired with opioid agonist therapy programs, though once more considerable heterogeneity exists among studies [52, 53]. A recent systematic review examining pharmacy-based needle/syringe exchange programs specifically appeared to show an effect in reducing risky behaviours among their clients, though measurable outcomes on HIV and HCV transmission could not be assessed in this context [54].

Lastly, safe injection sites are locations where clients who use illicit substances intravenously can access comprehensive health services including clean injecting equipment, nursing support, and referral to addiction treatment if requested. In other contexts, such facilities have been shown to reduce transmission of infections and/or drug overdoses in a cost-effective manner, while also increasing access to addiction treatment [55]. To our knowledge, such a program has never been studied in the setting of a community pharmacy.

A variety of other evidence-based harm reduction approaches exist and have previously been implemented in environments such as community clinics and public health facilities, but never trialed in pharmacy settings. Point-of-care drug checking, either on-site at events or in the form of independent take-home fentanyl test strips, have both been examined with positive, albeit limited results [56]. In other settings this form of drug checking been shown to have good uptake and may lead to changes in dangerous drug use behaviours, though data is limited [57, 58]. Increasing access to test strips via community pharmacies would be a relatively easily implemented intervention, with the potential to be paired with naloxone distribution programs. The distribution of free “safe injection kits” and/or “safe smoking kits” (providing all the necessary clean, safe equipment to inject or smoke drugs), has also been a strategy implemented to reduce the chance of transmitting infections, but has not been studied in rigorous fashion even outside the pharmacy context [59]. Given the limited evidence it is difficult to recommend these approaches be adapted to community pharmacies, but opportunities for further research are present.

## Dispensing of medications for the treatment of opioid use disorder

Opioid agonist therapy (OAT) with methadone or buprenorphine remains the most effective, evidence-based approach for the treatment of OUD. An extensive body of research supports the effectiveness of OAT at decreasing the use of illicit substances, at reducing criminal activity, at preventing transmission of bloodborne infections, and at protecting against overdose death [60]. Despite this evidence, a minority of patients with OUD in the US are currently treated with OAT [2]. While the reasons for this discrepancy are many, a significant barrier continues to be the physical access to both OAT prescribers and the medications themselves [61].

The most commonly used form of OAT used in the US is buprenorphine, however administration of buprenorphine requires regular visits to a DEA-waivered buprenorphine prescriber, and access to these clinics may depend very much on geographic setting [62]. Rural patients, in particular, can be subject to prohibitively long travel distances in order to access a physician who can prescribe this medication [63]. Less than half of US counties have a waivered physician, and of these counties without a buprenorphine provider, 82% were in rural America [64]. Increasing the number of DEA-waivered buprenorphine prescribers in the US is thus a priority, and in one physician survey 54% of non-prescribers suggested that they would be willing to prescribe if some of the mandatory requirements around prescription of buprenorphine were decreased [65]. The passage of the Comprehensive Addiction and Recovery Act (CARA) in 2016 partially addresses this deficiency by expanding DEA waiver status to nurse practitioners and physician assistants, but the need remains significant. Insufficient time, lack of space, and difficulties accessing pharmacies were all identified as barriers contributing to non-prescribing status, suggesting that further engaging pharmacies in the process of administering the medication could be one strategy to help expand the pool of prescribers [65]. Unfortunately, while buprenorphine is an effective medication, as many as 40% of those started on buprenorphine are not successfully retained in treatment at 6 months, thus increasing access to second-line options is still necessary [66].

Methadone is the other form of OAT most commonly used in the US, and has been used successfully in the treatment of OUD since the 1960s [67]. Retention rates for methadone vary substantially based on the dosing strategy, but compared to buprenorphine it is at least as effective at retaining patients in treatment at 6 months [66, 68]. Access to this medication is even more problematic than buprenorphine, however, given that federal law mandates it be administered exclusively via opioid treatment programs (OTPs)—designated, accredited facilities providing comprehensive care for patients with OUD [69]. Current estimates are that there are approximately 1500 OTPs within the US, offering methadone treatment to 356,000 clients [62]. Furthermore, while the opioid crisis has disproportionately affected rural America, 96% of all OTPs in the US are located within urban areas, meaning access for non-metropolitan populations can be extremely limited [70, 71]. There remain three states without any OTPs at all, and another four states with three or less operating OTPs [61]. Despite the dramatic rise in the prevalence of OUD over the past two decades, the number of OTPs in operation has remained more or less stable since 2003 [72]. There are also insurance coverage related barriers to accessing care through OTPs. Methadone for OAT is not a covered benefit for many state Medicaid programs or the US Medicare program. Effective 2020, the US Medicare program will provide coverage for methadone treatment through OTPs.

In contrast to the US model, a number of countries around the world have adopted a pharmacy-based approach to assist with the distribution of methadone as OAT. Examples of countries who use this approach include Canada, Australia, and the United Kingdom. In these systems, a patient is initially assessed by either an addiction specialist or a primary care provider, who then sends them to their local pharmacy with a prescription for the administration of their daily witnessed ingestion of OAT. They continue to visit their pharmacy daily to receive their dose of medication, with intermittent scheduled visits back to their prescriber to assess dose and provide additional care as needed. This was, in fact, the process for administering methadone when it was first used in the US, before OTPs were mandated in 1972 [69]. In contrast to OTPs, this approach offers much more flexibility in terms of accessing methadone, especially for patients in rural settings. In the US, for instance, the estimated number of pharmacies is 67,000, compared to 1500 OTPs [62, 73]. The cost of such a model is also comparable to the US system, with estimated prices of approximately €2–24 per patient per day in the UK [74], $11 per patient per day in Australia [75], $15 per patient per day in Canada [76], and $13 per patient per day in the US [77]. It is important to note that such a model would not necessarily replace OTPs, which specialize providing comprehensive care for patients who require it. In one Canadian study comparing community pharmacies to integrated treatment centres, the administration of methadone directly at the health care centre improved retention in treatment by > 45%, highlighting the need to be able to select from a variety of approaches depending on the individual circumstances of each patient [78].

While using pharmacies to assist with the distribution of methadone is clearly a strategy to improve geographic access, a number of stakeholders stand to be impacted by such a policy change, including the clients, the public, and the pharmacy staff themselves. Studies examining the perspectives of these various stakeholders have been undertaken, driven by potential concerns including the risk of medication diversion, the possibility of increased substance use and/or crime in the areas surrounding participating pharmacies, the perceptions of the general public using community pharmacies, and the safety of the pharmacy staff.

Despite these hypothetical concerns, numerous studies in participating countries have demonstrated that a significant number of pharmacists believe the provision of OAT from community pharmacies is an important service for them to provide [79–81]. Consequently, pharmacy participation in such programs has been high. In Scotland, 79% of pharmacies are actively involved in the distribution of methadone [82]. In England these numbers are very similar, with 79% of pharmacies indicating that they either currently dispense OAT (either methadone or buprenorphine), or would be willing to [81]. In each of these countries the attitudes of pharmacists towards their role in providing substitution therapy have been increasingly positive over time, and in one study nearly 25% of pharmacists were interested in further expanding their role in the provision of services for this population [81]. Methadone dispensing numbers are slightly lower in Australia, with less than 40% of pharmacies participating, yet among those who were dispensing their satisfaction with the program was excellent, with 98% indicating high levels of support [79, 80]. The most common issues reported by participating pharmacists were around payments, intoxicated patients, and occasional theft and/or patient aggression [79, 83]. Opinions on the prevalence of medication diversion vary widely, with anywhere from 30 to 74% of OAT dispensers believing that this was a significant issue [80, 84]. In multiple settings, difficulties communicating with the primary prescriber have also been identified as a frustration experienced by participating pharmacists, and the importance of strategies to enable pharmacist–physician communication in these partnerships is key for both patient safety as well as pharmacist satisfaction [83, 85].

While comparatively less studied, the attitudes of OAT clients receiving their medications directly from pharmacies have also been generally positive. In one Australian study, less than 15% of clients reported that they were dissatisfied with their current treatment, though one issue that was raised consistently was concern around patient confidentiality when receiving their doses [86]. Another US study surveyed a group of people who inject drugs and asked them what services they would like to see brought to community pharmacies. The provision of methadone through pharmacies rather than OTPs was among the top suggestions, with convenience and clinic hours being highlighted as putative benefits of this type of change [87]. Again, however, confidentiality was raised as a potential concern with such an approach.

Lastly, despite the international prevalence of pharmacy-based OAT dispensing models, little work has been done to measure the opinions of the general public on this approach to treatment. In one study, members of the public in Scotland were interviewed regarding their opinions on the availability of methadone and other harm reduction services through local pharmacies. Significant concerns were expressed around their own personal comfort and safety, as well as perceived ineffectiveness of these measures. Frequent stereotyping and stigmatization of methadone patients was a consistent theme noted in this qualitative analysis [88]. These study results are unfortunately consistent with population level surveys on drug treatment policy in the country around the same time, reflecting the ongoing need for public education while making efforts to improve individual access to addiction treatment [89].

In addition to OAT, extended-release naltrexone is a third medication for the treatment of opioid use disorder that is now available in the US. While the data support its effectiveness in the reducing opioid use, in practice it has been less effective than methadone or buprenorphine. This is due at least in part to a tendency for premature discontinuation of the medication, with 6-month retention rates as low as 10% based on retrospective review [90]. The reasons for this are likely many, though linkage to continuing care and the logistics of administering doses have been identified as contributors [91]. Minimizing any barriers to receiving a monthly dose could conceivably encourage longer retention, especially in patients where difficulties with access may be playing a role in the early discontinuation. At least 21 US states currently grant privileges to pharmacists for the administration of non-vaccine injectable medications, including extended-release naltrexone [92]. While pharmacy remuneration, education, consistency in state law, and client awareness have all been identified as barriers in helping pharmacies administer injectable medications, anecdotal reports of community pharmacies establishing extended-release naltrexone programs for patients with OUD do exist [93, 94].

Finally, there are several medications used elsewhere in the treatment of severe, refractory OUD that are not currently available in the US. These include formulations of slow-release oral morphine (SROM), as well as injectable opioid agonist therapy (iOAT) with either intravenous diacetylmorphine or hydromorphone. For patients who have not had success on methadone, the use of SROM is supported by moderate level evidence suggesting that it can both decrease illicit opioid use and reduce negative medication side effects [95]. The use of iOAT is supported by strong evidence demonstrating both a reduction in illicit opioid use and increased retention in treatment [96]. In Canada, for example, both of these treatment options are available for patients who have not had success on existing first- and second-line therapies, and can be dispensed through participating pharmacies [97, 98]. SROM is administered in a similar fashion to methadone and buprenorphine, with a prescription provided by an addiction medicine specialist being taken to a community pharmacy for daily witnessed administration. For iOAT, three models of delivery have been described: designated iOAT centers, integration of iOAT services into community clinics, or provision of iOAT through participating community pharmacies [98]. In the latter scenario, patients are initially titrated to a therapeutic dose of medication by a trained physician at their clinic. Once stable, they can be transferred to a participating pharmacy, where they attend in a similar manner as for other forms of OAT. The medication is dispensed two to three times daily, and patients then self-administer witnessed doses in a designated, private area within the pharmacy. In this model patients can be monitored by the pharmacy staff for signs of toxicity, and sent back to their prescriber for further dose adjustment if needed. Such a system of iOAT delivery has not been studied formally, but is currently being piloted across numerous centers in Canada.

## SUPPORT for Patients and Communities Act

In recent years, several important pieces of legislation (21st Century Cures Act, Comprehensive Addiction and Recovery Act) have substantially increased federal funding directed at the opioid crisis. As previously discussed, in October of 2018, President Donald Trump signed the Substance Use-Disorder Prevention that Promotes Opioid Recovery and Treatment (SUPPORT) for Patients and Communities Act, which contains several provisions relevant to pharmacists. The most directly applicable section (Chapter 2—Empowering Pharmacists in the Fight Against Opioid Abuse) instructs the Department of Health and Human Services, the Drug Enforcement Administration, the US Food and Drug Administration, and the Centers for Disease Control and Prevention to develop and disseminate materials for pharmacists, other healthcare providers, and patients on circumstances in which pharmacist may decline to fill controlled substance prescriptions. Input from boards of pharmacy, pharmacy associations, medical societies and licensing boards, healthcare providers, and patients will be solicited to support the development of these materials. Although it is unclear from the legislative language what specifically these materials will address, guidance of this type is sorely needed as pharmacists often express uncertainty in their role screening controlled substance prescriptions [99].

Pharmacists often express uncertainty in their role screening controlled substance prescriptions which, while legitimate, may be risky [24]. SUPPORT also contains a few provisions applicable to the Medicare program. By 2021, prescriptions covered by the Part D program for controlled substances must be transmitted electronically. Additionally, Medicare must provide for electronic prior authorization processes from providers to Part D plans for covered drugs. These provisions will likely streamline the controlled substance prescription verification process and reduce prior authorization-related administrative barriers for providers, pharmacists, and patients.

## Conclusions

North America has been severely affected by an increasing burden of opioid-related morbidity and mortality. The US response has been wide-ranging, including expanded funding for research, improved educational programs, and changes in health policy related to the treatment of substance use disorders. Despite these positive efforts, there remain resources that have yet to be leveraged to their maximum potential. Community pharmacists are ideally positioned for a role in surveying for signs of OUD, in contributing to programs designed to reduce the harms associated with opioid use, and in assisting with the treatment of patients with OUD. Low-hanging fruit include increasing access to tools and programs for OUD screening, facilitating patient education efforts, improving awareness of the role pharmacies can play in sterile syringe and naloxone distribution, and considering the adoption of legislation to enable pharmacy-based OAT distribution. Future research efforts must be directed towards the careful evaluation of how strategies successfully implemented elsewhere around the world might be adopted to an American context, however others are already well supported by data from pilot programs in place within the US. The current crisis necessitates a comprehensive response. Embracing new ideas such as these will assist in putting US pharmacists in the best position possible to help improve the overall care of patients with OUD.

## Data Availability

Data sharing not applicable to this article as no datasets were generated or analysed during the current study.

## Abbreviations

HCV: hepatitis C virus
HIV: human immunodeficiency virus
iOAT: injectable opioid agonist therapy
OAT: opioid agonist therapy
OTP: opioid treatment program
OUD: opioid use disorder
MTM: medication therapy management
PDMP: prescription drug monitoring program
SROM: slow-release oral morphine
